# Involvement of autophagy in ovarian cancer: a working hypothesis

**DOI:** 10.1186/1757-2215-5-22

**Published:** 2012-09-13

**Authors:** Claudia Peracchio, Oscar Alabiso, Guido Valente, Ciro Isidoro

**Affiliations:** 1 Laboratory of Molecular Pathology and Nanobioimaging, Department of Health Sciences, Novara, Italy; 2 Unit of Oncology, Department of Translational Medicine, Azienda Ospedaliero-Universitaria “Maggiore della Carità”, Novara, Italy; 3 Laboratory of Pathology, Department of Translational Medicine, Università del Piemonte Orientale “A. Avogadro”, Novara, Italy; 4 Department of Health Sciences, Università del Piemonte Orientale “A. Avogadro”, Via Solaroli 17, 28100, Novara, Italy

**Keywords:** Ovary cancer, Autophagy, Inflammation, Epigenetic, MicroRNA

## Abstract

Autophagy is a lysosomal-driven catabolic process that contributes to preserve cell and tissue homeostases through the regular elimination of damaged, aged and redundant self-constituents. In normal cells, autophagy protects from DNA mutation and carcinogenesis by preventive elimination of pro-oxidative mitochondria and protein aggregates. Mutations in oncogenes and oncosuppressor genes dysregulate autophagy. Up-regulated autophagy may confer chemo- and radio-resistance to cancer cells, and also a pro-survival advantage in cancer cells experiencing oxygen and nutrient shortage. This fact is the rationale for using autophagy inhibitors along with anti-neoplastic therapies. Yet, aberrant hyper-induction of autophagy can lead to cell death, and this phenomenon could also be exploited for cancer therapy. The actual level of autophagy in the cancer cell is greatly affected by vascularization, inflammation, and stromal cell infiltration. In addition, small non-coding microRNAs have recently emerged as important epigenetic modulators of autophagy. The present review focuses on the potential involvement of macroautophagy, and on its genetic and epigenetic regulation, in ovarian cancer pathogenesis and progression.

## Introduction

Ovarian cancer ranks as the fifth leading cause of cancer-related deaths among women, and the leading cause of death from gynecological cancer [1]. The difficulty to diagnose the disease at early stage and the persistence of dormant, drug-resistant cancer cells that cause relapse, are the primary reasons for the high mortality rate in ovarian cancer patients [2]. First-line therapy for advanced stage disease includes maximal surgical debulking followed by platinum/taxane chemotherapy, which attains initial response rates of over 80% [3]. However, most patients will eventually relapse with chemoresistant tumors. The propensity to trigger a program of epithelial-to-mesenchymal transition, the over-expression of drug efflux transporters and the persistence of dormant cancer stem cells are the principal factors that determine the recurrence and progression of ovarian cancer. The poor prognosis in ovarian cancer patients poses the urge to identify novel and more reliable (in terms of sensitivity and specificity) biomarkers for the detection of the disease in its (very) early stage, for monitoring the response to treatments, and possibly for targeted molecular therapy [4]. Recently, autophagy dysregulation in cancer cells has been blamed as a possible cause of dormancy and of resistance to radio- and chemotherapeutic treatments, and proteins involved in the regulation of this process are being considered as targets for anticancer molecular therapy. In this review, we discuss the involvement of (macro)autophagy in the pathogenesis of ovarian cancer, and on the genetic and epigenetic factors that potentially regulate this process. We also discuss the clinical implications of the role of autophagy in ovarian cancer for diagnosis, prognosis and therapy purposes.

### Morphology of autophagy at a glance

**Figure 1:**
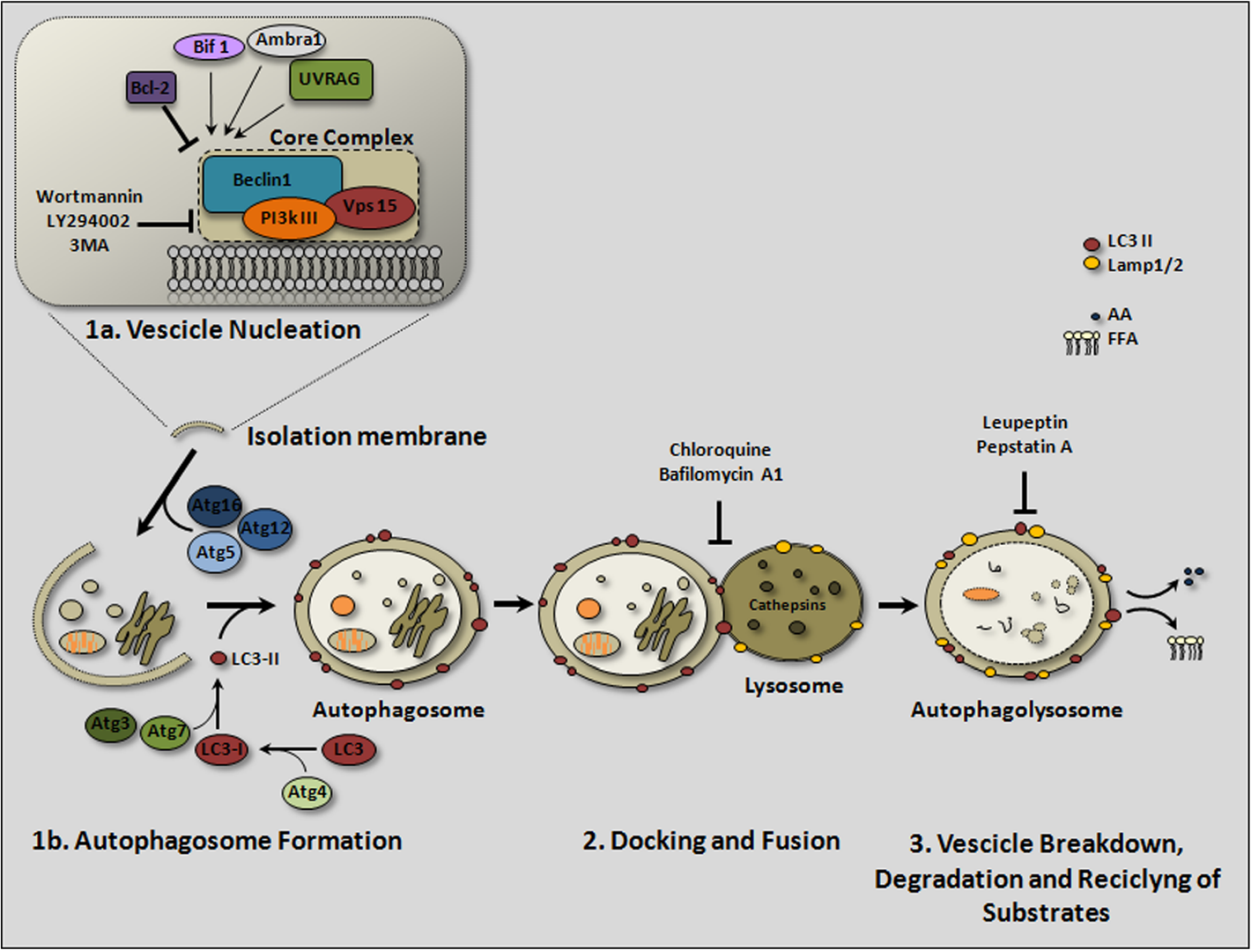
**Flow-chart showing the three principal steps of the (macro)autophagy process.** The first step starts with the vesicle nucleation from a pre-existing isolation membrane and terminates with the formation of an autophagosome that entraps cellular materials. The core complex of the autophagy interactome, and some other beclin 1 interactors, are shown in the inset. The kinase activity of PI3k class III can be inhibited by Wortmannin, LY294002 or 3-methyadenine (3MA). The interaction of bcl-2 with beclin 1 precludes the formation of the beclin 1-PI3k III complex. JNK-mediated phosphorylation of bcl-2 or DAPk-mediated phosphorylation of beclin 1 disrupts the bcl-2/beclin 1 interaction, and thus favors the formation of the autophagy interactome. During vesicle nucleation and expansion, a lipidated LC3-II isoform is included in both the internal and external membrane of the autophagosome. Atg4 plays a crucial role in the generation of LC3 II from LC3 I. Other Atg proteins (namely, atg3, atg5, atg7 and atg12) participate in the process of lipidation (i.e., conjugation with phosphatydil choline) and membrane insertion of LC3 II. The second step consists in the docking and fusion of the autophagosome with several endosomes and lysosomes to form the autophagolysosome. This step can be inhibited by drugs that increase the lysosome pH (e.g. Chloroquine, Bafilomycin A1). The third step consists in the degradation of the autophagic vesicle and of its cargo by acid hydrolases, and subsequent release of substrates (essentially AA, aminoacids; FFA, free fatty acids) for reutilization. The lysosomal degradation step can be inhibited by protease inhibitors (e.g., Leupeptin, Pestatin A) or by raising the internal pH.

Autophagy literally means (from Greek) ‘self-eating’, and refers to a cellular process committed to the lysosomal degradation of self constituents [5]. So far, three different types of autophagy (macroautophagy, microautophagy and chaperon-mediated autophagy) have been described, which essentially differ for the mechanism through which the target substrates gain access to the lysosomal lumen. In the case of macroautophagy (now on simply referred to as autophagy), macromolecular aggregates, portion of cytoplasm, membranes and entire organelles are sequestered within newly formed vesicles (named autophagosomes) that subsequently fuse with lysosomes [6]. In the case of microautophagy, cytoplasmic material and organelles are directly internalized by the lysosome through invagination of the lysosomal membrane [7]. In the case of chaperon-mediated autophagy, cytoplasmic proteins bearing the consensus sequence KFERQ at the C-terminus are assisted to enter the lysosome by the chaperon Hsc70, which interacts with the lysosomal membrane protein Lamp2A [8]. Schematically, three main operational steps characterize the autophagy process (Figure 1): (1) sequestration of the material into a newly formed vesicle; (2) fusion of this vesicle with lysosomal organelles; and (3) degradation of the material and recycling of the substrates. These steps have been widely characterized at morphological level [9], and new guidelines for their assessment have been recently released [10]. The hallmark of autophagosome formation is represented by the insertion within the inner and outer layers of the vesicle of LC3 II (isoform II of Light Chain), which is generated from the precursor Microtubule Associated Protein (MAP-LC3) by partial proteolysis and subsequent lipidation at its C-terminus [11]. The fusion of the autophagosome with late endosomes and lysosomes can be assessed by co-labeling LC3 and Lamp1 (the latter is a Lysosomal Associated Membrane Protein). Another means to look at the autophagy flux is to follow the degradation of p62/SQSTM1, a protein that links ubiquitinated protein aggregates to LC3 [12]. Once the autophagolysosome has formed, acid hydrolases (particularly, the cathepsins) degrade the sequestered material, and the substrates are recycled for biosynthetic processes [13,14].

### Biochemical regulatory aspects of autophagy

**Figure 2:**
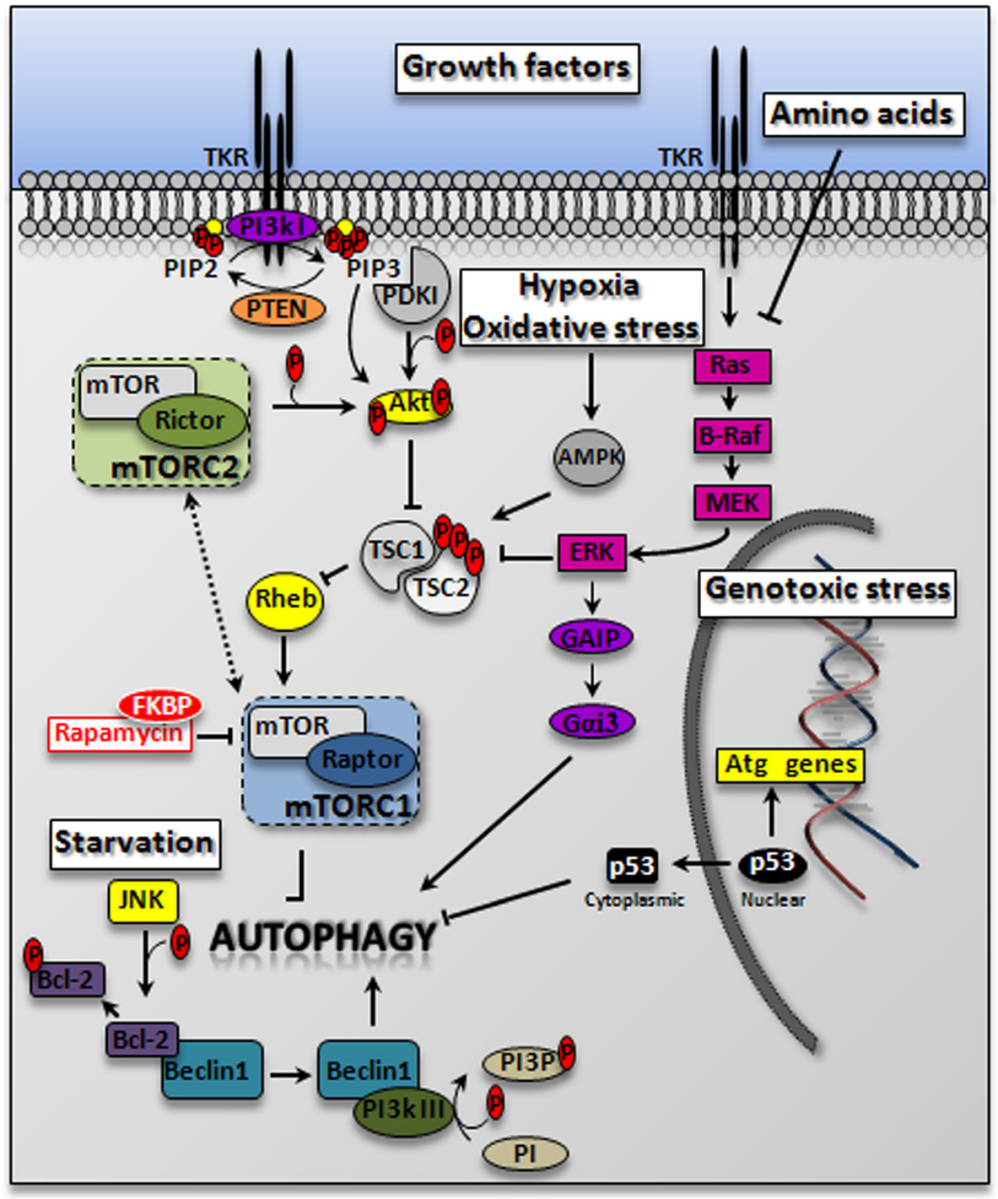
**Signaling pathways impinging on autophagy.** The scheme illustrates the network of the principal kinases involved in the regulation of autophagy. Essentially, growth factors activate the mTORC1 complex (trough the inhibition of the TSC1/TSC2 complex), and this results in the inhibition of the ULK1(ATG1) complex and prevents the induction of autophagy. The abundance of aminoacids also results in down-regulation of this pathway through the inhibition of the Ras-Mek-Erk pathway, and by direct activation of mTOR through the RagA/B GTPases complex (not shown in the scheme). The lipid-kinase activity of PTEN prevents the activation of the Akt-mTOR pathway, and therefore removes the tonic inhibition of autophagy exerted by this pathway. This tonic inhibition can be also removed artificially by pharmacologic inhibition of mTOR with Rapamycin. Hypoxia and mitochondrial oxidative stress inhibit mTOR through the action of AMPk on the TSC1/TSC2 complex. Starvation (amino acid deprivation) activates the JNK pathway, which ends with the phosphorylation of bcl-2, thus allowing the formation of the autophagy interactome. Once activated, Vps34 produces PI3P, which acts as a platform for other autophagy proteins involved in autophagosomal membrane nucleation and elongation. A genotoxic stress activates p53 and other transcription factors (e.g., TFEB) that promote the synthesis of autophagy proteins (e.g., DRAM, UVRAG, cathepsin D). However, high levels of cytoplasmic p53 (localized in the endoplasmic reticulum) result in the inhibition of autophagosome formation.

The biochemical regulation of autophagy has been the subject of excellent recent reviews, to which the readers may refer for a detailed description [15,16]. A variety of protein- and lipid-kinases, protein- and lipid-phosphatases, and mono and trimeric GTPases control the induction and progression of autophagy [15-19]. A simplified network of the main regulatory pathways is illustrated in Figure 2. Classically, the starting signal for the formation of the autophagosome is the synthesis of phosphatydil-inositol-3-phosphate (PI3P) molecules by the PI3k class III kinase (also known as Vps34), which becomes active upon interaction with Beclin 1 (homologue of Vps30/Atg6) [20], see also Figure 1. By contrast, the production of phosphatydil-inositol-3,4,5-phosphate (PIP3) by class I PI3k keeps basal autophagy at low level through the activation of the Akt pathway [21]. The lipid phosphatase activity of the oncosuppressor PTEN, which removes the phosphate in position 3 from PIP3, counteracts the activation of Akt and therefore allows autophagy [22]. A crucial player in the regulation of autophagy is mTOR (mammalian Target of Rapamycin), whose kinase activity inhibits Atg1/ULK1 and the formation of the autophagy interactome [18]. While the Akt pathway negatively regulates autophagy through the activation of mTOR, the AMPk pathway, which senses the lack of ATP, inhibits mTOR and directly activates Atg1 (homologue of ULK1), thus promoting autophagy in response to stressors [23]. It follows that activation of autophagy may occur in the presence of phosphorylated Akt, provided that mTOR is inactivated [24]. It is to be mentioned that autophagy may also be induced bypassing the mTOR control, for instance by increasing the level of Inosytol-tri-phosphate (IP3) [25]. Remarkably, at variance of the canonical pathway described above, mammalian cells can activate alternative pathways for autophagic degradation in which certain autophagy proteins (e.g., Beclin-1, Vps34, Atg5, Atg7, ULK1) are dispensable (reviewed in [26]).

Simplifying, amino acids and growth factors keep mTOR active and prevent the hyper-induction of autophagy, whereas nutrient shortage and energy depletion increase the level of basal autophagy (Figure 2).

### Functional significance of autophagy in normal and in cancer cells

Autophagy accomplishes two fundamental physiological activities: (1) it constitutively degrades and recycles redundant and aged molecules and organelles, and (2) it destroys abnormal and potentially harmful molecular and cellular components [15]. To understand the role of autophagy in cell physiology and pathology it is fundamental to distinguish basal and induced autophagy. Basal (constitutive) autophagy prevents unwanted and unnecessary increases in cell mass by eliminating exuberant and exceeding cellular structures, thus greatly contributing to cell homeostasis. In normal cells, constitutive autophagy is subjected to modulation depending on the metabolic state of the cell: while in growing cells active mTOR exerts a tonic inhibitory control that keeps constitutive autophagy at low basal level, in normal quiescent cells constitutive autophagy is up-regulated to equilibrate synthesis in order to allow the macromolecular turnover without net increases in cell mass. On demand, autophagy can be transitorily hyper-induced, for instance to meet the need to recover from nutrient or energy depletion, or to contrast the accumulation of damaged molecules and organelles. In cancer cells autophagy is clearly dysregulated. The strict connection between autophagy and carcinogenesis is supported by the fact that numerous oncogene and oncosuppressor proteins regulate both processes [27]. Autophagy plays a complex and apparently contradictory role in the various phases of cancer development and progression. In fact, by avoiding the accumulation of damaged molecules and organelles that may increase the probability of oxidative stress-mediated DNA mutation, basal autophagy prevents cell transformation [28]. Consistently, loss-of-function of genes that positively regulate autophagy, such as Beclin-1 [29,30] or PTEN [31], predisposes to spontaneous cancers. On the other hand, when a chemotherapeutics induces oxidative stress and DNA damage or when defective vascularization determines hypoxia and starvation, the up-regulation of autophagy enables cancer cells to overcome the metabolic stress [32,33]. In such circumstances, up-regulation of autophagy associated with down-regulation of apoptosis contributes to chemoresistance. Further, a long-lasting up-regulation of basal autophagy may favor a senescent/dormancy state in cancer cells [34], and likely in cancer stem cells as well, that resist to radio- and chemotherapy, and could be re-activated and give rise to recurrent cancer.

### Epigenetic regulation of autophagy: the role of histone deacetylases, of microRNAs, and of the tumor microenvironment

Besides the fact that autophagy can be dys-regulated as a consequence of mutations in autophagy-related genes, including oncogenes and oncosuppressor genes, the possibility that the actual level of autophagy in cancer cells is dynamically influenced by epigenetic factors should be taken into account. The principal intrinsic epigenetic regulation occurs at transcriptional level, through modulation of DNA methylation and histone acetylation, and at post-transcriptional level through microRNA(miRNA)-mediated degradation of mRNA. For instance, the expression of ARH1 gene, an oncosuppressor that regulates autophagy (see below), is repressed in many cancers, including ovarian cancers, due to hyper-methylation of its promoter [35]. Moreover, certain miRNAs, a class of small non-coding RNAs, have recently emerged as important epigenetic modulators of autophagy in cancer cells (reviewed in [36]). The mRNA of several autophagy-related genes contains, in fact, the target sequence for miRNAs belonging to different families, with either oncosuppressive or oncogenic activities. For instance, Beclin-1 mRNA can be targeted by oncosuppressive members of the miR30 family [37] and by oncogenic miR-376b [38]; the mRNA of ATG4C is down-regulated by oncogenic miR-376b [38] and that of ATG4D by oncosuppressive miR-101 [39]; the mRNA of p62/SQSTM is targeted by miR-17/20/93 and miR106 [40], while the expression of MAP-LC3 can be affected by miR196 [41], and the translation of ATG7 mRNA is suppressed by miR375 [42]. Finally, autophagy can be modulated through modulation of hystone acetylation/deacetylation by the hystone deacetylases HDAC4 and HDAC5, which are targeted by miR-9-3p [43]. To be noted, HDAC6 promotes the autophagic flux by regulating the acetylation status of cytoskeleton proteins [44].

Additional mechanisms of epigenetic regulation involve extracellular signals. In fact, microenvironmental factors (hypoxia, pH, oxidative stress, nutrient availability, cytokines, hormones and growth factors) and the physical-metabolic interaction with surrounding cells (inflammatory cells, fibroblasts) in the matrix greatly influence the autophagy compliance of the tumor cell [45-47]. The scarce vascularization in the most inner portion of the tumor determines a situation of hypoxia and starvation, which cause the activation of autophagy driven by the Hypoxia Inducible Factor HIF-1α [48] and by AMPk [49], thus conferring a survival advantage to cancer cells. On the other hand, in the highly vascularized area of the tumor, the presence of nutrients limits autophagy and favors the growth of cancer cells. In addition, cancer associated fibroblasts, inflammatory cells (especially type-2 macrophages) and cytokines (e.g., IL-1β, TNF-α and IL-6) have been shown to affect the regulation of autophagy in cancer cells through induction of a metabolic stress [47,50-52].

### Involvement of autophagy in ovarian cancer

According to genetic and pathologic features, epithelial ovarian cancer are classified in Type I tumors, characterized by a variety of somatic mutations or amplification/deletion of oncogenes or oncosuppressors including K-RAS, B-RAF and PTEN, and Type II tumors, which are chromosomally unstable, and present with mutated or deleted TP53 (in more than 80% of the cases) and BRCA inactivation (in up to 30% of the cases) [53,54]. Type-1 ovarian cancers comprise clinically indolent, low grade serous and endometrioid carcinomas, clear cells and mucinous carcinomas; type-2 ovarian cancers comprise aggressive, high-grade serous and endometrioid carcinomas, malignant mixed mesodermal carcinomas, and undifferentiated carcinomas [53,54]. Besides the genetic alterations, it is now becoming clear that also epigenetic mechanisms play a role in the development of ovarian cancer [55]. Yet, the mechanisms involved in epithelial ovarian cancer pathogenesis and progression are still largely obscure. Autophagy dysregulation might play an important role in the pathogenesis, as well as in resistance to radio- and chemotherapeutic treatments and in dormancy in ovarian cancer. Indeed, a number of oncogenes and oncosuppressor genes have been found deregulated in ovarian cancers because of genetic or epigenetic alterations [56,57], and many of these potentially impact on autophagy regulation. Furthermore, a plethora of proteins whose expression has been found altered in ovarian cancers may directly or indirectly affect autophagy at different level. Schematically, the genes found altered in ovarian cancer that have an impact on autophagy belong to: (1) the oncosuppressors PTEN, ARHI and p53, that regulate autophagy, apoptosis and dormancy; (2) the components of the autophagy machinery LC3, beclin-1 and DRAM; (3) the growth factor and nutrient sensor signaling pathways, which include the class I PI3-k/Akt/mTOR and the Ras/Raf/ERK pathways.

**Figure 3:**
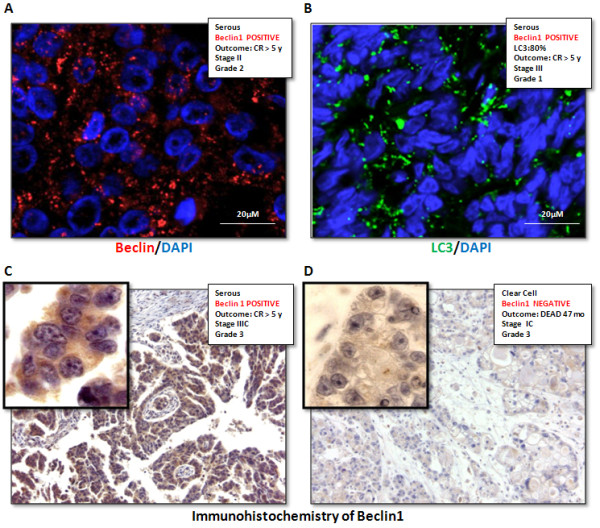
**Immunodetection of autopahgy proteins in ovarian cancer tissue sections.** Immunofluorescence positivity for beclin 1 (panel **A**) and LC3 (panel** B**) appears as discrete puncta (indicative of protein clusterization) in the cytoplasm of cancer cells. A high level of expression of beclin 1 and of LC3 (positivity in > 40% of cancer cells) was found to correlate with good prognosis (CR, complete remission at 5 years of follow up). Immunohistochemistry of beclin 1 in a positive (panel **C**) and in a negative (panel **D**) case is also shown (magnification 420x). The presence or absence of beclin 1 aggregates can be appreciated in the high-magnification (1050x) area shown in the insets.

As compared to benign hyperplastic tissues and borderline ovarian tumors, poorly differentiated and highly malignant ovarian cancer cells were shown to express very low level of the autophagy protein LC3 [58], indicating that LC3-labeled autophagosomes do not accumulate in highly aggressive ovarian cancers. Whether this phenomenon underlies the inability to form autophagosomes or rather reflects their efficient removal by the lysosomal system remains to be elucidated. Mutation and deletion of the oncosuppressor P53 gene has been reported in 60–80% of both sporadic and familial ovarian cancers [56]. DNA-binding deficient p53 mutants are unable to sequester bcl-2 or bcl-XL, and display a dominant negative activity. Bcl-2 can inhibit the formation of the autophagy interactome by interacting with beclin 1 [20], and therefore the over-expression of such mutated p53 in ovarian cancer cells may indirectly impact on autophagy. In addition, p53 mutants that permanently localizes in the cytoplasm have been shown to inhibit autophagy [59]. ARH1 (Aplasia Ras Homolog member I; also known as DIRAS3), which encodes a ras-homolog 26 kDa GTPase, is a tumor suppressor gene imprinted down-regulated in ovarian cancers [60]. Re-expression of ARH1 suppresses proliferation, motility and angiogenesis [61,62] and promotes cell death [63] in ovarian cancer cells. Of note, ARH1 protein has recently been shown to up-regulate autophagy (through inhibition of the mTOR pathway) and to induce autophagy-dependent dormancy in ovarian cancer cells [34]. The latter finding implies that re-activation of ARHI can enable ovarian cancer cells to overcome metabolic stress and to survive in a dormant state in appropriate tumor microenvironment. As stated above, active mTOR exerts a tonic inhibition on basal autophagy. It is intriguing, in this respect, the finding that a hyper-active status of mTOR is associated with a poor prognosis in ovarian carcinoma patients [64]. Sustained up-regulation of the class I PI3k-Akt-mTOR axis in ovarian cancers may arise from activating mutation or duplication of genes coding for the Tyrosin Kinase Receptors EGFR and PDGFR, for PI3kCA or Akt [65,66], as well as by inactivating mutations of PTEN [67] or hyper-expression of the PTEN-regulator protein DJ-1 [68]. BECLIN 1 has been the first oncosuppressor gene that proved the link between autophagy and cancerogenesis [69]. Of note, monoallelic deletion of BECLIN 1 is found in more than 50% of sporadic ovarian cancers [69]. Accordingly, the expression of beclin 1 was found down-regulated in ovarian cancers, compared to benign lesions [58]. Consistent with a role of autophagy-active beclin 1 in ovarian cancer progression, we found that hyper-expression of both beclin 1 and LC3 in ovarian cancer cells was associated with a good chemotherapeutic response in patients (Peracchio et al., unpublished data; Figure 3). Another autophagy-regulator gene associated with ovarian cancer progression is DRAM (Damage-Regulated Autophagy Regulator), a p53-trancribed gene that codes for a lysosomal-associated protein involved in apoptosis and autophagy [70]. Recently, it has been reported that the homologous DRAM2, which also induces autophagy [71], is expressed at very low level in aggressive ovarian tumors [72]. Transgenic over-expression of PEA-15, the 15 kDa Phospho-Enriched protein in astrocytes, has recently been shown to induce autophagy and non-apoptotic cell death in ovarian cancer cells through the activation of the ERK pathway [73]. It is intriguing to observe that women bearing an ovarian cancer with high level of expression of PEA-15 have an overall survival longer than those bearing a low-PEA15-expressing cancer [73]. In aggregate, the data so far available consistently indicate that an intrinsic defect in the activation of autophagy leads to a more aggressive progression of ovarian cancer.

### Epigenetic factors that impinge on autophagy in ovarian cancer

As mentioned above, autophagy in cancer cells is subjected to fluctuations depending on extracellular stimuli, the availability of oxygen and nutrients, and also on the actual expression of certain microRNAs. Ovarian cancer cells release chemotactic cytokines and growth factors that recruit fibroblasts, endothelial cells and macrophages, which in turn contribute with their own secretions to form a dynamic tumor microenvironment [74,75]. A number of inflammatory-related proteins abnormally present in the tumor context or in the ascitic fluid, and associated with ovarian cancer progression, could directly or indirectly affect autophagy. For instance, TNFα, a cytokine involved in ovarian cancer growth and metastasis [74], is a potent activator of NF-kB, which in turn activates the anti-apoptotic and anti-autophagic Akt/mTOR pathway. IL-6 is a pro-inflammatory cytokine highly expressed in the tumor context of type-2 ovarian cancers and in ascitic fluid, and its level correlates with poor prognosis in ovarian cancer patients [76,77]. IL-6 promotes VEGF-mediated vasculogenesis and angiogenesis, especially in aggressive type-2 ovarian cancers [77]. Lysophosphatidic acid (LPA), abundantly released by ovarian cancer cells, is known to contribute to ovarian cancer aggressiveness by stimulating the synthesis of IL-6 and of VEGF [78,79], among others. Of note, while IL-6 acts as an inducer [80], LPA was shown to inhibit autophagy induced by serum deprivation in prostate cancer cells [81]. These data outline how the microenvironment and the cytokine network dynamically affect autophagy in ovarian cancer cells. In the epigenetic control of autophagy, a further level of complexity is brought by the dynamic changes in the expression of miRNAs. The profile of miRNAs pattern in ovarian cancer cells varies during development and progression phases [82-84]. Although a thorough analysis of miRNA-mediated regulation of autophagy in ovarian cancer cells has not yet been performed, we can speculate in this sense based on the information available. For instance, miR-30a, which negatively regulates the expression of Beclin 1 [37], was found down-regulated in samples from relapsing patients diagnosed with stage I ovarian cancer [85]; and miR-101, which represses the expression of the autophagy protein Atg4 [39], was found down-regulated in ovarian cancer compared to normal tissue [86]. MiR-101 targets also the mRNA of STMN1 and RAB5A [39]. Of note, stathmin over-expression showed a significant association with poor prognosis in ovarian cancer patients [87], and Rab5A was shown to promote cell proliferation in ovarian cancer [88]. Finally, miR-214 and mir-21, respectively associated with the chemoresistant phenotype [89] and the metastatic potential of ovarian cancer cells [90], have been shown to target PTEN, the oncosuppressor known to positively regulate autophagy and to be mutated or deleted in a vast majority of ovarian carcinomas. With regard to the transcriptional level of epigenetic regulation of autophagy in ovarian cancer, the oncosuppressors PTEN, ARH1 and DAPk (Death-associated protein kinase) merit to be mentioned. The hyper-methylation of PTEN promoter is not a frequent finding in ovarian cancer specimen [91]. By contrast, ARH1 and DAPk are among the most frequently down-regulated tumor suppressors in ovarian cancers due to promoter methylation [35,91]. Under stressful conditions, DAPk phosphorylates beclin-1, promoting its dissociation from bcl-2, and thus inducing autophagy [92].

### Clinical implications and future perspectives

Targeting of the autophagy pathway is being under evaluation as a new anti-cancer therapeutic option [93-95]. Data in the literature show that both autophagy enhancer and autophagy inhibitor drugs may elicit beneficial effects by inducing cancer cell death. This apparent contradiction could be explained considering the complex role that autophagy plays in cancer cells in the different phases of carcinogenesis, and in dependence of the tumor context. In fact, while at the precancerous stage an autophagy defect would facilitate genomic instability and tumor development, in growing tumors the up-regulation of autophagy compensates for the limited nutrient supply and helps to face genotoxic and metabolic stresses [96,97]. The latter phenomenon constitutes the rationale for using inhibitors of the late step of the autophagy process (e.g., chloroquine) together with traditional anti-neoplastics [93-95]. In certain circumstances, the excessive and sustained up-regulation of autophagy (for instance under prolonged starvation, oxidative stress or metabolic impairment) has been associated with cell death [98,99]. This observation constitutes the rationale for using drugs that induce autophagy, such as rapamycin and its analogs [93-95]. The switch from a pro-survival to a pro-death outcome of autophagy activation could also be exploited in cancer therapy. Relevant to the present study, a phase II clinical trial for the treatment of endometrioid ovarian cancer with an mTOR inhibitor is currently ongoing [100]. At present, 22 clinical trials (of which 2 are terminated) are using rapamycin or its analogs in combination with other drugs for the treatment of ovarian cancer ( http://www.clinicaltrials.gov). Another drug that has been proposed as a molecular therapeutics for the treatment of ovarian cancers is the anticonvulsant Valproic acid (VPA), which acts as a histone-deacetylase inhibitor [4]. At present, 2 ongoing clinical trials are using VPA alone or in combination with Carboplatin for the treatment of ovarian cancer ( http://www.clinicaltrials.gov). VPA and tubacin (another histone deacetylase) kill ovarian cancer cells at doses that specifically inhibit HDAC6 [101], which is known to favor the fusion of autophagosomes with lysosomes through the deacetylation of tubulin and actin [44]. Thus, targeting autophagy might be a strategy to combat ovarian cancers that have developed chemoresistance to traditional antiblastic therapies. However, it must be kept in mind that the efficacy of autophagy-based therapies strictly depends on the actual level of ongoing autophagy in the tumor cells, which is dictated by genetic mutations, but also influenced by the epigenetic regulation of relevant genes, as mediated by the tumor microenvironment (namely the vascularization and the extent of the infiltration by fibroblasts and immuno-inflammatory cells) and by certain miRNAs. The unraveling of clinical implications of genetic and epigenetic factors involved in autophagy dysregulation in ovary cancer might hopefully open the way to new diagnostic and therapeutic approaches for this malignant disease.

## Competing interests

The authors declare that they have no competing interests.

## Authors' contributions

CP performed the immunofluorescence staining, made the bibliography search and drew the schemes; GV performed the immunohistochemistry; GV and OA participated in the critical assessment of the manuscript. CI organized the structure of the review and wrote the manuscript. All authors read and approved the final manuscript.
